# The History, Status, Gaps, and Future Directions of Neurotoxicology in China

**DOI:** 10.1289/ehp.1409566

**Published:** 2016-01-29

**Authors:** Tongjian Cai, Wenjing Luo, Diyun Ruan, Yi-Jun Wu, Donald A. Fox, Jingyuan Chen

**Affiliations:** 1 Department of Occupational and Environmental Health, Ministry of Education Key Lab of Hazard Assessment and Control in Special Operational Environment, School of Public Health, Fourth Military Medical University, Xi’an, Shaanxi, China; 2 Department of Epidemiology, College of Preventive Medicine, Third Military Medical University, Chongqing, China; 3 Neurotoxicology Lab, School of Life Science, University of Science and Technology of China, Hefei, Anhui, China; 4 Laboratory of Molecular Toxicology, Institute of Zoology, Chinese Academy of Sciences, Beijing, China; 5 College of Optometry,; 6 Department of Biology and Biochemistry,; 7 Department of Pharmacological and Pharmaceutical Sciences, and; 8 Department of Health and Human Performance, University of Houston, Houston, Texas, USA

## Abstract

**Background:**

Rapid economic development in China has produced serious ecological, environmental, and health problems. Neurotoxicity has been recognized as a major public health problem. The Chinese government, research institutes, and scientists conducted extensive studies concerning the source, characteristics, and mechanisms of neurotoxicants.

**Objectives:**

This paper presents, for the first time, a comprehensive history and review of major sources of neurotoxicants, national bodies/legislation engaged, and major neurotoxicology research in China.

**Methods:**

Peer-reviewed research and pollution studies by Chinese scientists from 1991 to 2015 were examined. PubMed, Web of Science and Chinese National Knowledge Infrastructure (CNKI) were the major search tools.

**Results:**

The central problem is an increased exposure to neurotoxicants from air and water, food contamination, e-waste recycling, and manufacturing of household products. China formulated an institutional framework and standards system for management of major neurotoxicants. Basic and applied research was initiated, and international cooperation was achieved. The annual number of peer-reviewed neurotoxicology papers from Chinese authors increased almost 30-fold since 2001.

**Conclusions:**

Despite extensive efforts, neurotoxicity remains a significant public health problem. This provides great challenges and opportunities. We identified 10 significant areas that require major educational, environmental, governmental, and research efforts, as well as attention to public awareness. For example, there is a need to increase efforts to utilize new in vivo and in vitro models, determine the potential neurotoxicity and mechanisms involved in newly emerging pollutants, and examine the effects and mechanisms of mixtures. In the future, we anticipate working with scientists worldwide to accomplish these goals and eliminate, prevent and treat neurotoxicity.

**Citation:**

Cai T, Luo W, Ruan D, Wu YJ, Fox DA, Chen J. 2016. The history, status, gaps, and future directions of neurotoxicology in China. Environ Health Perspect 124:722–732; http://dx.doi.org/10.1289/ehp.1409566

## Introduction

This review is based on proceedings of the International Neurotoxicology Conference held in Xi’an China June 2011. The purpose of the Conference was to review the current status of neurotoxicology, identify research gaps, and make recommendations concerning future directions for neurotoxicology in China. The use of neurotoxicants in China can be traced to 3000 BCE (Table 1). In 1975, a bronze sword from ~ 3000 BCE was found in Gansu Province. From then until the Tang Dynasty (618–907), Chinese bronze, an alloy of copper, tin, and lead, was used in cooking and food containers. Early treatises (200–600 CE) focused on mushroom-induced neurotoxicity and treatments (Table 1). In 1593, lead and tetrodotoxin neurotoxicity, and updated herbal treatments for mushroom-induced neurotoxicity were described (Table 1). In the 1980s, modern neurotoxicology research in China started when neurobehavioral tests were implemented to protect occupational workers. In 1986, the Neurobehavioral Core Test Battery (NCTB), translated into Chinese, was introduced (Chen et al. 1999). In 1988, a computer-based Chinese version of the Neurobehavioral Evaluation System (NES-C1) was established. The NES-C1 was updated in 1992 and 1998 (Chen et al. 1999). In the 1990s, laboratory-based mechanistic studies, epidemiology studies, biomarker studies, and neuroprotective studies of neurotoxicants were initiated.

**Table 1:** Chronicle of major neurotoxicology events in China from 3000 BCE to 2014 AD.

Date	Events
3000 BCE	Bronze use began in China (Lee et al. 2008)
200 CE	Zhongjing Zhang described the symptoms and treatment of mushroom-induced neurotoxicity in *Synopsis of Prescriptions of the Golden Chamber* (Zhang 2012)
610 CE	Yuanfang Chao further described mushroom-induced neurotoxicity in *General Treatise on the Cause and Symptoms of Diseases* (Chao 2009)
1593 CE	Shizhen Li described the toxicity of lead, use of herbal medicines against mushroom-induced toxicities, and neurotoxicity of tetrodotoxin from globefish in *Compendium of Materia Medic* (Li 2005)
1965 CE	Chelating therapy for lead intoxications was introduced into China (Wang et al. 1965)
1979 CE	Environmental Protection Law approved for trial implementation (National People’s Congress 1979)
1982 CE	First edition of Ambient Air Quality Standard issued (SEPA 1982); Marine Environment Protection Law adopted (National People’s Congress 1982)
1984 CE	Law on Prevention and Control of Water Pollution adopted (National People’s Congress 1984)
1985 CE	First edition of Standards for Drinking Water Quality released (MOH 1985)
1986 CE	WHO Neurobehavioral Core Test Battery (NCTB) introduced into China (Chen et al. 1999)
1987 CE	First edition of Law on the Prevention and Control of Atmospheric Pollution enacted (National People’s Congress 1987)
1988 CE	Computer-based Chinese Version of the Neurobehavioral Evaluation System (NES-C1) was formed (Chen et al. 1999)
1989 CE	Environmental Protection Law amended and formal one adopted (National People’s Congress 1989); six cases of chronic manganese intoxication in workers at a ferromanganese factory reported (Huang et al. 1989)
1995 CE	Law on Prevention and Control of Environmental Pollution by Solid Waste adopted (National People’s Congress 1995b); second edition of Law on the Prevention and Control of Atmospheric Pollution adopted (National People’s Congress 1995a)
1996 CE	Second edition of Ambient Air Quality Standard released (SEPA 1996); second edition of Law on Prevention and Control of Water Pollution adopted (National People’s Congress 1996)
1999 CE	Second edition of Marine Environment Protection Law adopted (National People’s Congress 1999)
2000 CE	Third edition of Law on the Prevention and Control of Atmospheric Pollution adopted (National People’s Congress 2000); use of leaded gasoline banned (He et al. 2009)
2002 CE	Indoor Air Quality Standard of China released (SEPA 2002)
2004 CE	Second edition of Law on Prevention and Control of Environmental Pollution by Solid Waste adopted (National People’s Congress 2004)
2006 CE	Second edition of Standards for Drinking Water Quality released (MOH 2006b); the “Trial Implementation Guide to The Classification and Treatment Principles for Child-Related High Blood Lead Levels and Lead Poisoning Cases” was released (MOH 2006a)
2008 CE	China upgraded State Environmental Protection Administration (SEPA) to be Ministry of Environmental Protection (MEP) (Qiu and Li 2008); third edition of Law on Prevention and Control of Water Pollution adopted (National People’s Congress 2008)
2010 CE	The Chinese Neurotoxicology Association (CNA) established (Chinese Society of Toxicology 2010)
2011 CE	Xi’an International Neurotoxicology Conference (XINC) held (Fox et al. 2012; Zheng 2012)
2012 CE	Third edition of Ambient Air Quality Standard released (MEP 2012)
2013 CE	Third edition of Law on Prevention and Control of Environmental Pollution by Solid Waste adopted (National People’s Congress 2013a); third edition of Marine Environment Protection Law adopted (National People’s Congress 2013b)
2014 CE	Second edition of Environmental Protection Law adopted (National People’s Congress 2014)

This paper reviews the major sources of neurotoxicants, the history of national bodies, and regulations and legislation related to neurotoxicity, major neurotoxicology research institutes and organizations, and papers describing research on selected neurotoxicants in China.

## Methods

Peer-reviewed research and pollution studies by Chinese scientists from 1991 to 2015 were examined. PubMed, Web of Science and Chinese National Knowledge Infrastructure (CNKI) were the major search tools.

## Results

### Major Sources of Neurotoxicants in China

China’s recent economic expansion is one of the strongest in world history (Kan et al. 2012). However, accelerated urbanization and industrialization has increased the release of numerous toxicants and neurotoxicants, and produced numerous adverse ecological, environmental, and health problems (Kan et al. 2012; Ahearn 2011). Approximately 2.4 million deaths in China per year are attributed to the poor environmental quality (Zhang and Xu 2011).

***Air pollution.*** Air pollution is a major exposure pathway of neurotoxicants. In China, fine particulate matter (PM_2.5_, particles with aerodynamic diameters ≤ 2.5 μm) is the largest contributor to the air pollution (Wang et al. 2014). Particulate matter (PM) has strong potential for absorbing toxic metals, which makes heavy metals, a family of neurotoxicants, the important components of PM (Li H et al. 2013). Compared with coarse PM, PM_2.5_ has a greater surface area per unit mass, allowing it to accumulate heavy metals more effectively (Li H et al. 2013). It is also more poisonous than coarser PM because of its longer residence in air and deeper penetration into lungs (Li H et al. 2013). Globally, 1.6 million premature deaths per year are associated with indoor air pollution; of these, 420,000 are in China (Mestl and Edwards 2011). Although improved, China still has the worst air pollution in the world (Kan et al. 2009). As a result of industrialization, urbanization, and increased vehicle use, air pollution occurs in major cities (Chen B et al. 2011). Coal constitutes ~ 75% of energy sources in China and outdoor air pollution predominantly consists of coal smoke (Kan et al. 2009). Decreased childhood exposure to polycyclic aromatic hydrocarbons emitted from coal-burning plants in China was associated with improved neurobehavioral development (Perera et al. 2008). Indoor air pollution is another leading environmental health risk, as ~ 70% of Chinese households burn coal or biomass for cooking and heating (Millman et al. 2008). Tobacco made in China contains high levels of heavy metals (O’Connor et al. 2010) and tobacco smoke is a large source of indoor air pollution (Salo et al. 2004). Unregulated chemicals and neurotoxicants used in the manufacturing of toys, floors, and furniture also contribute to indoor air pollution. For example, polybrominated diphenyl ethers (PBDEs), a family of brominated flame retardants (BFRs) with known developmental neurotoxicity effects (Costa et al. 2014), are widely used in numerous household products, with the domestic demand increasing at a rate of approximately 8% per year in China (Ni et al. 2013).

***Water pollution.*** Polluted water is another ubiquitous exposure pathway to neurotoxicants. For instance, China has the greatest industrial use of mercury, a typical heavy metal, and leads to the elevated water pollution in China (Lin et al. 2012). China has ~ 20% of the world’s population, but only 8% of its fresh water (Beach 2001). About 700 million Chinese drink water that does not meet the Chinese Standards for Drinking Water Quality (Beach 2001). From 2000 to 2008, 6,677 water pollution accidents occurred in China threatening the safety of water sources (Zhang XJ et al. 2011). For example, the explosion of an aniline production factory in 2005 resulted in the discharge of more than 100 tons of nitrobenzene and related compounds into the Songhua River, the fourth longest river in China, forcing Harbin, a city with four million inhabitants, to be out of water for 4 days (Li et al. 2008). For groundwater, neurotoxic pesticides and fertilizers seep underground and pollute the only available source of drinking water for millions, especially in rural areas where dependence on well water is absolute (Beach 2001). For surface water, heavy metals from mining-related industries, and the extensive use of fertilizers from farmlands are major sources of pollution (Zhang and Shan 2008; Zhang X et al. 2012). Increased shipping and industrial wastes contribute heavy metals to waterways (Ye et al. 2011).

***Food contamination.*** Food safety problems attracted increased public attention in recent years. Improper use of agrochemicals, fertilizers, and pesticides in agriculture all threaten the primary food production (Lam et al. 2013). In China, food safety is threatened by the contamination of heavy metals and pesticides (Lu et al. 2015). In farming areas either adjacent to lead and zinc mines (Li et al. 2006) or using wastewater on soils (Xue et al. 2012), fruits and vegetables contain high levels of heavy metals. In the Pearl River Estuary, high concentrations of cadmium were found in crab, shrimp and shellfish samples and of lead in fish (Ip et al. 2005). In Nanjing, ~ 97% of breast milk samples had lead levels > 5 μg/L, the limit set by the World Health Organization (WHO) (Liu KS et al. 2013; Parr et al. 1991).

Due to the large population and relatively small arable farmland, pesticides are used extensively to increase agricultural yield (Hu et al. 2015). Approximately 10% of rice samples in China contain detectable residues of organophosphate pesticides (OPs) (Chen et al. 2009). In Xiamen, ~ 20% of cabbage, legumes, and leaf mustard had pesticide residues exceeding maximum residue limits (MRLs) allowed by Chinese regulations (Chen C et al. 2011). In Shaanxi Province, mean levels of omethoate, phorate, chlorpyrifos, methidathion, and ethoprophos residues in vegetables exceeded MRLs (Wang S et al. 2013).

***E-waste recycling.*** Uncontrolled e-waste recycling–induced pollution is of global concern (Yang et al. 2012). Contamination exists in a number of locations in China, especially South China (Luo et al. 2011). High levels of polybrominated biphenyls (PBBs), polybrominated diphenyl ethers (PBDEs), persistent organic pollutants polychlorinated biphenyls (PCBs), polychlorinated dibenzo-*p*-dioxins and dibenzofurans (PCDD/Fs), and heavy metals from the e-waste recycling processes were detected in tissue and blood samples from children and neonates (Song and Li 2014). In areas surrounding primitive e-waste processing facilities, the soil and vegetables contain high levels of neurotoxicants (Luo et al. 2011).

***Manufacturing of household products.*** After decades of economic expansion, China is a worldwide producer of daily household products such as toys and stationeries (Weidenhamer 2009). In 2007, most of the toys recalled in the USA for lead contamination were manufactured in China (Weidenhamer 2009). Bisphenol A (BPA), a potential neurotoxicant (Perera et al. 2012), is an important industrial chemical primarily used as an intermediate in the production of polycarbonate plastics and epoxy resins, which are widely used in digital media, electronic equipment, automobiles, construction glazing, sports safety equipment, medical devices, tableware, reusable bottles (e.g., baby bottles) and food containers (Huang et al. 2012). The demand and production capacity of BPA in China have grown rapidly (Huang et al. 2012).

### National Agencies and Legislation of Major Toxicants in China

***National agencies.*** In China, the National Health and Family Planning Commission (NHFPC) [former Ministry of Health (MOH)] and the Ministry of Environmental Protection (MEP) are the major governmental bodies responsible for environmentally related neurotoxicology issues. In March 2013, China established the NHFPC by merging MOH with NHFPC. For environmental areas, the responsibilities of NHFPC are to draft health standards and supervise their enforcement; conduct health education; develop programs on prevention and treatment of diseases; and organize comprehensive prevention and treatment of major pollution-related diseases. Upgraded from State Environmental Protection Administration (SEPA) in 2008 (Qiu and Li 2008), the MEP is a cabinet-level ministry charged with protecting China’s air, water, and land from pollution and contamination and is required to implement environmental policies and enforce environmental laws and regulations. The Chinese Center for Disease Control and Prevention (CCDC) is an agency of the NHFPC. Its predecessor was the Chinese Academy of Preventive Medicine founded in 1983 and was renamed trans-CCDC in 2002. CCDC focuses national attention on developing and applying disease prevention and control, environmental health, occupational safety and health, health promotion, and prevention and education activities.

***Legislation and regulation of major toxicants.*** The Environmental Protection Law was approved for trial implementation in 1979 (National People’s Congress 1979) and was amended in 1989 (National People’s Congress 1989) and 2014 (National People’s Congress 2014). In 1987, the Law on the Prevention and Control of Atmospheric Pollution was enacted (National People’s Congress 1987): amended in 1995 (National People’s Congress 1995a) and 2000 (National People’s Congress 2000). Several other environmental laws such as the Law on Prevention and Control of Environmental Pollution by Solid Waste [adopted in 1995 (National People’s Congress 1995b); amended in 2004 (National People’s Congress 2004) and 2013 (National People’s Congress 2013a)], the Law on Prevention and Control of Water Pollution [adopted in 1984 (National People’s Congress 1984); amended in 1996 (National People’s Congress 1996) and 2008 (National People’s Congress 2008)], and the Marine Environment Protection Law [adopted in 1982 (National People’s Congress 1982); revised in 1999 (National People’s Congress 1999) and 2013 (National People’s Congress 2013b)] were formulated. Furthermore, the Criminal Law (National People’s Congress 1997) provides detailed measures for the penalty of criminals leading to environmental pollution in Article 338 and 339. Whoever causes severe environmental pollution through the discharging of pollutants, or import and disposition of overseas solid pollutants, shall be sentenced to imprisonment up to 10 years with/without fines (National People’s Congress 1997).

Mostly drafted by MOH and MEP, China formulated its own environmental standards system. In 1982 (SEPA 1982), the national Ambient Air Quality Standard was issued and amended in 1996 (SEPA 1996) and 2012 (MEP 2012). There are standards for 10 pollutants: sulfur dioxide, total suspended particulates, 2.5 and 10 μm inhalable particulate matter, nitrogen oxides, nitrogen dioxide, carbon monoxide, ozone, fluoride, lead and benzo[*a*]pyrene. In 2002, the Indoor Air Quality Standard was released: 19 indexes were included and carbon monoxide was the only neurotoxicant (SEPA 2002). In 1985, the first edition of Standards for Drinking Water Quality was released with 35 indexes including six heavy metals: arsenic (0.05 mg/L), cadmium (0.01 mg/L), copper (1.0 mg/L), lead (0.05 mg/L), manganese (0.1 mg/L) and mercury (0.001 mg/L) (MOH 1985). In 2006, the second edition increased the number of indexes from 35 to 106 and had two different types of standards: one for common centralized water supply projects, the other for small or non-centralized water supply projects. In the former, the limits decreased for arsenic (0.05 to 0.01 mg/L), cadmium (0.01 to 0.005 mg/L) and lead (0.05 to 0.01 mg/L), and aluminum (0.2 mg/L) was added. In the latter, the limits were 0.05 mg/L for arsenic and 0.3 mg/L for manganese, with no differences for aluminum, cadmium, copper, lead and mercury (MOH 2006b).

Codex Alimentarius Commission, created by the Food and Agriculture Organization (FAO) and WHO, established pesticides residues standards for agricultural products. The National Standards for MRLs of Pesticides in Food were issued in August 2014 by the NHFPC and Ministry of Agriculture (MOA) in China (Song et al. 2014). MRLs restrict the permitted concentration of a residue and type of commodity on which it is allowed. This new standard includes 3,650 MRLs for 387 pesticides in 284 different kinds of agricultural products and foods. MRLs are based on risk assessment using pesticide residue analysis data from market samples or appropriate supervised field trials and food consumption data. These MRLs are in compliance with internationally recognized food standards (Song et al. 2014).

### Overview of Neurotoxicology Research by Chinese Authors

**Figure 1:**
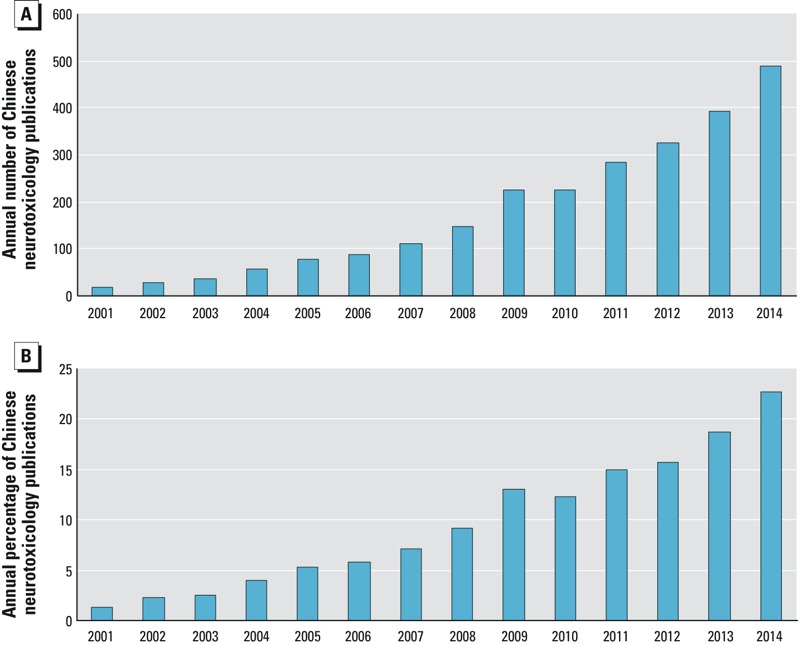
Peer-reviewed neurotoxicology papers published from 2001 through 2014 by Chinese authors. (*A*) Annual number of Chinese neurotoxicology publications. (*B*) Worldwide annual percentage of neurotoxicology papers published by Chinese authors. Data were obtained from Web of Science TM Core Collection with the Citation Indexes as Science Citation Index Expanded (SCI-EXPANDED) and Social Sciences Citation Index (SSCI) up to 1 January 2015 with co-application of the following strategies: topic was neurotoxicity, authors’ addresses were in China, and document types were peer-reviewed articles and reviews.

Using the Web of Science TM Core Collection with the Citation Indexes as Science Citation Index Expanded (SCI-EXPANDED) and Social Sciences Citation Index (SSCI), we searched for peer-reviewed original papers or reviews published in international journals with co-application of the following strategies—topic was neurotoxicity, and authors’ addresses were in China (at least one author was from China). From 2001 to 2014, there were 23,235 papers published on the subject of neurotoxicology worldwide and 10.8% of those papers were written by Chinese authors. The annual number of papers from Chinese authors increased from 17 in 2001 to 488 in 2014 (Figure 1A) or 1.4% and 22.7% of the total number of papers for those years, respectively (Figure 1B), indicating that Chinese scientists were actively engaged in international neurotoxicology research.

### Highlights of Major Neurotoxicology Research Areas

Due to space limitations, only highlights on major neurotoxicants will be presented. These studies contributed important new information on the sites/mechanisms of and potential neuroprotection from major neurotoxicants. Research on the neurotoxicity of brominated flame retardants, polycyclic aromatic hydrocarbons, solvents, some biotoxins and electromagnetic fields will not be discussed.

### Heavy Metals: Lead, Manganese, Mercury, Aluminum and Arsenic

***Lead.*** Globally, China is one of the largest lead producers and consumers of lead (Zhang X et al. 2012). The main sources of lead pollution in China are ore and metal processing, manufacturing, and combustion of coal, petroleum fuel, and wastes (Cheng and Hu 2010). Childhood lead poisoning is a major public health problem in China (Zhang SM et al. 2009). Although blood lead levels decreased after its use in gasoline was banned (July 2000), mean blood lead levels of Chinese children is still higher than in developed countries (He et al. 2009). Lead pollution from e-waste recycling and tinfoil processing also is a threat to children (Wang X et al. 2012).

Developmental lead exposure produces cognitive, behavioral, auditory, retinal, and visual-motor dysfunction as well as neuropsychiatric alterations (Canfield et al. 2003; Fox and Boyes 2013; Goyer 1993; Nagpal and Brodie 2009; Osman et al. 1999; Rothenberg et al. 2000, 2002; Wasserman et al. 2000). Many Chinese scientists contributed to this research and helped elucidate sites and mechanisms of lead neurotoxicity. For example, they found that lead exposure produced hearing loss (Liu S et al. 2011) and retinal ganglion cell dysfunction (Ruan et al. 1994) in rats. Wang Q et al. (2011a) reported that *in vitro* lead is transported through the blood–brain barrier by a divalent metal transporter 1 IRE-positive isoform, which can be inhibited by iron (Wang Q et al. 2011a). Others showed that blood delta-aminolevulinic acid dehydratase (ALAD) activity (Wang Q et al. 2011b) and its polymorphisms (Gao et al. 2010) were susceptibility biomarkers for lead neurotoxicity.

Synaptic plasticity plays a critical role in learning and memory and its impairment plays a critical role in lead neurotoxicity. Chinese scientists found that postnatal lead exposure produced age-dependent alterations in the induction of long-term depression and potentiation (Ruan et al. 1998; Xu et al. 1998), paired-pulse facilitation (Ruan et al. 1998), and short-term depression (Ruan et al. 2000) in rat hippocampus. Moreover, lead induced thyroid dysfunction (Wu et al. 2011); impaired calcium flux (Yan et al. 2008); altered ion channels (Gu et al. 2005); inhibited neural cell adhesion molecules (NCAMs) and sialyltransferase activity (Hu et al. 2008); and alterated neurotransmitters and metabolites (Sheng et al. 2005; Tang HW et al. 1996; Tang M et al. 2009), which likely contribute to synaptic plasticity impairments.

Chinese scientists found that lead altered activation of Ca2+/calmodulin-dependent enzymes (Zhang GS et al. 2012) and extracellular–regulated protein kinase (ERK) signaling (Zhang Y et al. 2007); altered methylation patterns of amyloid precursor (Li YY et al. 2012) and ALAD genes (Li C et al. 2011); increased tau phosphorylation and beta amyloid (Li et al. 2010); induced oxidative stress (Zhang YM et al. 2009), inflammation (Li N et al. 2009), and endoplasmic reticulum Ca2+ release (Fan et al. 2013); and decreased nitric oxide (NO) (Sun L et al. 2005).

Treatment and prevention of lead poisoning remains a major health problem worldwide (Bazrgar et al. 2015). In 1965, chelation therapy was initiated in China. In 2006, MOH issued two official documents: “Guide to the Preventive Measures Against Child-Related High Blood Lead Levels and Lead Poisoning” and “Trial Implementation Guide to the Classification and Treatment Principles for Child-Related High Blood Lead Levels and Lead Poisoning Cases” in which chelation therapies by meso-2,3-dimercaptosuccinic acid (DMSA) and calcium disodium ethylenediamine tetraacetic acid (CaNa_2_EDTA) are major treatment measures (MOH 2006a). However, both agents have potential risks: DMSA can lead to gastrointestinal discomfort, skin reaction, mild neutropenia, and elevated liver enzymes while CaNa_2_EDTA can lead to renal failure, arrhythmias, tetany, hypocalcaemia, hypotension, bone marrow depression, prolonged bleeding time, convulsions, and respiratory arrest (Flora and Pachauri 2010). Chinese scientists examined alternative novel therapeutic strategies. Various drugs and herbs partially or totally rescued lead-induced neurotoxicity, such as omega-3 fish oil (Cao et al. 2010), methionine choline (Fan G et al. 2010), hippophae rhamnoides L. juice (Xu et al. 2005), selenium (Liu MC et al. 2013), puerarin (Liu CM et al. 2013), ginsenoside Rd (Wang B et al. 2013), tea catechins (Chen et al. 2003), and iron (Wang Q et al. 2007).

***Manganese.*** Due to high industrial use and low self-protection, there are many people affected by chronic manganese toxicities in China (Wang Y et al. 2012). Clinical studies found that long-term manganese exposure to welders was associated with impaired brainstem parasympathetic and sympathetic centers receiving axon projections from cortical and diencephalic areas (He and Niu 2004), and changes in mood, behavior, and peripheral neurotransmitters (Yuan et al. 2006). Susceptibility to manganese-induced neurotoxicity is influenced by a CYP2D6L gene polymorphism (Zheng et al. 2002). Furthermore, laboratory studies have reported that manganese neurotoxicity was related to enhanced oxidative stress (Xiao et al. 2009; Zhang S et al. 2004); reduced mitochondrial enzyme activity (Zhang S et al. 2003); proteasome dysfunction (Cai et al. 2007); and nuclear localization and subsequent binding of NF-E2-related factor 2 (Nrf2) to the antioxidant-responsive element (ARE); and/or upregulation of heme oxygenase-1 protein (Li H et al. 2011). Manganese also adversely affected astrocytes (Deng et al. 2011; Fan X et al. 2010); activated microglia (Zhao et al. 2009); increased tau hyperphosphorylation and α-synuclein expression (Cai et al. 2010, 2011); increased extracellular glutamate and inhibited expression of its *N*-methyl-D-aspartate (NMDA) receptor subunits in rat striatum (Xu B et al. 2010); increased [Ca2+] (Xu et al. 2009); induced p21 expression (Zhao et al. 2012b); and disrupted the Glu-Gln cycling (Deng et al. 2009). Interestingly, riluzole, taurine, dextromethorphan, para-aminosalicylic acid and increased dietary fiber antagonized manganese-induced neurotoxicity (Deng et al. 2012; Jiang et al. 2006; Shi et al. 2012; Xu Z et al. 2010).

***Mercury.*** Mercury adversely affects neurodevelopment (Llop et al. 2012). China contributes ~ 28% of global mercury emissions (> 600,000 kg per year) (Pacyna et al. 2006), which increased 164% from 1992 to 2007 (Liang et al. 2013). Consumption of methylmercury contaminated rice is the main source of mercury exposure (Zhang J et al. 2010). In Songyuan City (Jilin Province), 17% of the residents’ hair contains mercury > 1 mg/kg (reference dosage value set by U.S. EPA) (U.S. EPA 1997) even after the closure (20 years ago) of the acetic acid plant responsible for local mercury pollution (Zhang and Wong 2007). Gestational exposure to low doses of inorganic mercury (HgCl_2_) selectively increased hippocampal and cerebellar mercury levels (Feng et al. 2004). Mercury-induced neurotoxicity was associated with oxidative stress-dependent c-fos and c-jun expression (Cheng et al. 2005, 2006) (in rats fed by rice cropped in mercury-polluted farmlands) and tau protein aggregation (*in vitro*) (Yang et al. 2010). In fish, mercuric chloride impaired the development of the hypothalamic serotonergic system (Tsai et al. 1995).

***Aluminum.*** High brain levels of aluminum are neurotoxic and cause learning and memory deficits in laboratory animals (Cui et al. 2012; Struys-Ponsar et al. 1997). Chinese aluminum electrolytic workers have altered motor coordination, mood and parasympathetic nervous function (He et al. 2003). Experimental results suggest that aluminum-induced deficits in learning and memory result from altered synaptic configuration (Jing et al. 2004), activation/inactivation of ion currents of hippocampal CA1 neurons (Zhang B et al. 2004), enhanced function of nACh receptors (Hu et al. 2007), altered mitochondrial structure/function (Niu et al. 2005), decreased activities of kinases involved in LTP induction and formation (Wang et al. 2010), disturbed trace metal homeostasis (Yang et al. 1998), oxidative stress (Ding and Yang 2010) and/or apoptosis of cortical neurons and primary astrocytes (Fu et al. 2003; Guo and Liang 2001).

Neuroprotection studies found that vasopressin (Wang et al. 2001), naloxone (Sun SL et al. 2005), ginkgo biloba extract (Gong et al. 2005), meloxicam (Yang et al. 2006), caffeic acid (Yang et al. 2008), zinc (Song et al. 2008), tetrahydroxy stilbene glucoside (Luo et al. 2009), and ginsenoside Rb1 (Zhao et al. 2013) differentially prevented aluminum-induced neurotoxicity. Dipsacus asper (Zhang ZJ et al. 2003), gastrodia elata (He et al. 2008), and icariin (Luo et al. 2007) improved learning and memory in aluminum-intoxicated rats. Biochemical/genetic inactivation of Bcl-2 antagonist/killer (BAK) and caspase-3 delayed the onset of apoptosis in aluminum-treated cells (Zhang QL et al. 2009, 2010) suggesting the therapeutic potential of RNAi-based methods against aluminum-induced neurodegeneration.

***Arsenic.*** Arsenic is released into the atmosphere during coal processing and combustion (Kang et al. 2011). In China, approximately 520, 21 and 250 tons of arsenic are emitted annually by industries, residential buildings and coal-fired power plants, respectively (Kang et al. 2011). In Shanyin County (Shanxi province), arsenic exposure was associated with impaired children’s intelligence and growth (Wang SX et al. 2007). Experimental studies showed that arsenic exposure produced hippocampal ultrastructural changes, down-regulation of NMDA receptor and postsynaptic signaling (Luo et al. 2012), and inhibited hippocampal neurogenesis (Liu et al. 2012). Arsenic also modulated DNA methylation and contributed to neural tube defects via epigenetic mechanisms (Han et al. 2011), promoted nitrative DNA lesions (Piao et al. 2011), and down-regulated mitochondrial succinate dehydrogenase subunit A (Hong et al. 2009) and Camk4 (Wang et al. 2009). Neuroglobin (Ngb) had a protective role in the cerebellum against arsenite-induced oxidative stress (Wang J et al. 2012). Arsenic exposure resulted in lower brain nitric oxide synthase (NOS) activity and levels (Wang Y et al. 2011), and inhibited glutamate metabolism in astrocytes (Zhao et al. 2012a), which could impair synaptic formation (Wang Y et al. 2013).

### Fluoride

Fluoride exposure has been associated with altered intelligence in children (Tang et al. 2008) and it is prevalent throughout China (Chen et al. 2014). Animal studies have indicated that exposure to high concentrations of fluoride can affect performance in learning and memory tasks (Gui et al. 2010; Jiang S et al. 2014). Exposure to high concentrations of fluoride was associated with inhibited brain glucose utilization (Jiang C et al. 2014). It also down-regulates NCAMs (Zhang M et al. 2007), synaptic membrane fluidity (Zhu et al. 2011), and postsynaptic density protein-95 (Zhu et al. 2011) in hippocampus. On the other hand, fluoride exposure led to upregulated vesicle-associated membrane protein-2 (VAMP-2) (Han et al. 2014) in hippocampus and dysregulated intercellular Ca^2+^ *in vitro* (Xu Z et al. 2013; Zhang J et al. 2011). Other potential mechanisms include increased ERK1/2 (Liu et al. 2010), JNK (Liu YJ et al. 2011), and NF-κB (Zhang J et al. 2011) expression, microglia activation (Yan et al. 2013), abnormal mitochondrial dynamics (Lou et al. 2013), hippocampus glutamate alterations (Niu et al. 2009), and altered acetylcholine receptors and cholinesterase (Liu et al. 2010; Zhao and Wu 1998). Ginkgo biloba extract (Zhang et al. 2013) and selenium (Qian et al. 2013) had neuroprotective effects.

### Pesticides

In China, ~ 770 approved pesticides are on the market (Wu and Sun 2004). More than one million tons are used annually, ~ 60% are organophosphates (OPs) and ~ 20% are pyrethroids (Wang et al. 2008). Pesticide intoxication is a serious threat to human health as there are > 150,000 deaths per year from pesticide poisoning (Li Y et al. 2009). Pesticide poisonings account for ~ 20% of poisoning cases at emergency departments of 25 hospitals and have the highest fatality rate (5%) among all poisoning cases (Li Y et al. 2009).

### OPs and Carbamates

***OP-induced delayed neuropathy (OPIDN).*** OPIDN is the chronic neurotoxicity induced by OPs, characterized by distal axonopathy and progressive muscle weakness and flaccidity (Abou-Donia and Lapadula 1990; Glynn 2006). The underlying mechanism of OPIDN is complex and not fully understood. Suggested targets include cytoskeletal protein degradation (Chang and Wu 2006; Song et al. 2009), neuropathy target esterase (Chang and Wu 2006; Hou et al. 2009) and calcium homeostasis (Wu and Leng 1997; Wu et al. 2007). Intentional or accidental exposure to a number of OPs including mipafox, omethoate, leptophos, trichlorphon, parathion, methamidophos, fenthion and chlorpyrifos caused OPIDN in humans (Abou-Donia and Lapadula 1990; Jokanović et al. 2011). However, it is not clear whether some of these pesticides directly cause OPIDN (Lotti and Moretto 2005). Although some OPs were banned in China (methamidophos and parathion), others are still widely used such as omethoate (Ding and Tian 2014).

***Typical OPs and carbamates.* Methyl parathion.** Although methyl parathion was banned in 2007, its residue persists (Chen et al. 2009). In zebrafish brain, methyl parathion-induced protein changes were identified by matrix-assisted laser desorption/ionization time-of-flight mass spectrometry (Huang and Huang 2011). Proteomics also identified changes in protein levels after joint exposure to cadmium and methyl parathion in zebrafish brain (Ling et al. 2012).

***Chlorpyrifos.*** In brain of common carp, chlorpyrifos treatment induced nitric oxide (NO) and inducible NO synthase (iNOS) and led to oxidative stress and brain tissue damage (Wang LL et al. 2013). A novel non-cholinergic mechanism, the hyper-phosphorylation of GSK-3β, may contribute to its cellular and behavioral (depression) neurotoxicity (Chen et al. 2012). Mancozeb is an organometallic dithiocarbamate fungicide. Potentiation of voltage-gated KCNQ2 potassium channels was found to be a possible neurotoxic mechanism for mancozeb (Li P et al. 2013).

***Pyrethroids.*** Besides being axonal excitotoxicants that block sodium channels, newer modes of action for pyrethroids have been determined by a number of Chinese research groups. For example, deltamethrin increased the activities of NOS and poly(ADP-ribose) polymerase (Wu and Liu 1999), and induced apoptotic cell death in rat brains (Wu and Liu 2000). Deltamethrin inhibited tyrosine hydroxylase activity and dopamine synthesis in the nigrostriatal pathway in SD rats (Liu et al. 2006). In PC12 cells, NF-E2 related factor 2 activation protected cells from deltamethrin-induced oxidative stress (Li et al. 2007). In zebrafish embryos, Cypermethrin induced oxidative stress and apoptosis via caspase activation (Shi et al. 2011), whereas fenvalerate produced brain morphological abnormalities and apoptosis (Gu et al. 2010).

***Other pesticides/herbicides.* Paraquat.** Microglia activation, astrocyte edema, and neuronal cells apoptosis were found to be typical neurotoxic signs of paraquat acute exposure in rat brain (Wu et al. 2013). Cyperquat (1-methyl-4-phenylpyridinium, MPP+), structurally similar to paraquat, was used to study the mechanisms and possible therapies for Parkinson’s diseases (Ruan et al. 2011; Xu X et al. 2013; Zhai et al. 2013; Zhou et al. 2013). Simvastatin (Xu X et al. 2013), catechins (Ruan et al. 2011), secalonic acid (Zhai et al. 2013), and 3-O-demethylswertipunicoside (Zhou et al. 2013) were found to be able to protect neuronal cells from MPP+-induced apoptosis in cultured cells.

**Rotenone.** A broad-spectrum pesticide, rotenone inhibits mitochondrial electron transport, induces oxidative damage and produces apoptosis of dopaminergic neurons in mesencephalic neuron/glia cultures (Wang XJ et al. 2011). The flavone Baicalein exerted *in vivo* and *in vitro* neuroprotective effects on rotenone-induced neurotoxicity (Li XX et al. 2012).

**Avermectins.** Avermectins are widely used parasiticides in human/veterinary medicine and as pesticides in agriculture/horticulture (Lasota and Dybas 1991). Chinese scientists found that subcytotoxic levels of two avermectin derivatives were neurotoxic in differentiating neuronal cells, which may result from the down-regulation of P-glycoprotein 1 pump and cytoskeletal proteins (Sun et al. 2010).

## Conclusions, Gaps and Future Directions

In recent years, Chinese neurotoxicology researchers significantly contributed to laboratory studies of major environmental and industrial neurotoxicants. This produced an increased number of peer-reviewed publications by Chinese scientists, especially those employing cellular/molecular, bioinformatic, electrophysiological, morphological, neurobehavioral, neurochemical, and neuroimaging methodologies. Major problems and research areas still need attention. For example, there are only a few epidemiological studies compared to laboratory experiments. To date, no nationwide investigation on the breadth and extent of pediatric or adult human lead or pesticide neurotoxicity exists. Although laboratory experiments explored protective measures against lead neurotoxicity, no clinical studies have been conducted. For laboratory studies, more attention was focused on high-dose or high-concentration related models, and less on the adverse effects of low-level exposures. Moreover, the current neurotoxicology studies lack the necessary connection between field studies and laboratory research.

Following our comprehensive analysis, we propose that the following additional efforts are needed:

Although environmental standards and laws were formulated, they need strengthening in accordance with international standards.
Increase implementation of the laws across all of China. This is especially important in regions where more attention is directed to economic development than environmental protection.
Increase efforts to utilize new *in vivo* and *in vitro* models. In China, rodents are the major experimental animals employed for neurotoxicity studies. Studies on alternative species such as zebrafish and c. elegans for screening neurological impairments and developmental neurotoxicology should be enhanced. As of January 2015, only 46 and 21 neurotoxicology studies from Chinese authors used zebrafish or C. elegans, respectively.
Determine the potential neurotoxicity and mechanisms involved in newly emerging pollutants, especially those with potential gestational/neonatal and childhood exposure. In 2008, melamine-contaminated infant formula caused urinary tract stone in 290,000 children in China (Chen 2009). Then animal studies found that melamine could induce cognitive impairment in rats (An et al. 2012).
Examine the additive and/or synergistic effects and mechanisms of mixtures or combination of neurotoxicants. For example, lead has synergistic neurotoxicity with arsenic (Rai et al. 2010), cadmium (Kim Y et al. 2013), ethanol (Flora et al. 2012), manganese (Kim et al. 2009) and benzo[*a*]pyrene (Qi et al. 2013).
Determine the cellular interactions between progenitor cells and differentiated neurons and glia. Reciprocal interactions between glia and neurons are essential for many critical functions in brain health and disease (Carnevale et al. 2007). Deciphering the reciprocal interactions provides novel insights in understanding molecular mechanisms in both physiological and pathological conditions (Eyo and Wu 2013; Kim KH et al. 2013).
Enhance research devoted to solving practical matters, such as determining the subclinical features of neurotoxicities, finding new biomarkers, determining the translational links between laboratory work and improving human health, and evaluating effective neuroprotective measures. To promote applied research in combination with laboratory studies, the National Natural Science Foundation of China and Ministry of Science and Technology should emphasize and increase funding for combined neurotoxicology field and laboratory studies as well as for preventative measures and biomarker systems. In 2012, the first such large new project entitled “The mechanisms of environmental lead exposure-induced brain development impairment in children” was granted to Professor Jingyuan Chen, supported by Major State Basic Research Development Program of China (973 Program) from the Ministry of Science and Technology.
Enhance international collaborations. Environmental pollution is a global problem that needs to be solved cooperatively. With the world’s largest population (~ 1.5 billion) and its heavy environmental pollution, China has various endemic disease-affected areas, such as endemic arseniasis (Li S et al. 2012). A recent Science report suggested that 19.6 million people are at risk of being affected by the consumption of arsenic-contaminated groundwater in China (Rodríguez-Lado et al. 2013).
Investigate neurotoxicity in the aged population. China has an increasing aged population that will develop neurodegenerative diseases. However, little work has examined the epidemiology, preventive measures, and susceptibility of neurotoxicity in the aged.
Increase health awareness and education of the public. Although Chinese scientists and institutions have published more papers recently (Figure 1) than 10 years before, little attention and effort were made to disperse this knowledge.

In conclusion, this paper reviews the major sources of neurotoxicants, history of national agencies and regulations/legislation related to neurotoxicity, major neurotoxicology research institutes and organizations, and papers describing research on selected neurotoxicants in China. Furthermore, non-Chinese neurotoxicologists significantly contributed, educated and inspired Chinese investigators and authorities, especially during the early stages of Chinese neurotoxicology research. These collaborative efforts between Chinese and foreign scholars are ongoing. Collectively, Chinese neurotoxicologists face great challenges and opportunities. We believe the prevention of human neurotoxicity is not only a scientific, but also a social obligation and problem. We will continue to work with the scientists worldwide to eliminate, prevent, and treat neurotoxicity.
